# Intraprocedural OCT monitoring of the immediate treatment response during indocyanine green angiography-guided laser therapy of teleangiectatic capillaries in diabetic macular edema

**DOI:** 10.1038/s41598-022-05950-0

**Published:** 2022-02-10

**Authors:** Felix Datlinger, Anja Datlinger, Andreas Pollreisz, Stefan Sacu, Ursula Schmidt-Erfurth, Peter Datlinger

**Affiliations:** 1 Department of Ophthalmology and Optometry, Medical University of Vienna, Vienna, Austria; 2 Ophthalmological Practice Datlinger, Sonnwendgasse 5, 7210 Mattersburg, Austria; 3 Vienna Clinical Trial Center, Medical University of Vienna, Vienna, Austria; 4 Laboratory for Ophthalmic Image Analysis, Medical University of Vienna, Vienna, Austria

**Keywords:** Diagnostic markers, Predictive markers, Diabetes complications

## Abstract

In this retrospective study the morphological response of teleangiectatic capillaries (TCs) to focal laser treatment and the functional and morphological outcome after Indocyanine green angiography (ICGA)-guided laser therapy was evaluated. TCs in eyes with diabetic macular edema (DME) were treated with laser therapy. The immediate and subsequent reaction of the TCs lumina to direct photocoagulation was monitored with customized OCT single scans. Additionally, patients were treated with intravitreal anti-VEGF as needed. 12 eyes of 9 patients with treatment naive (6 eyes) and pretreated (6 eyes) DME were followed-up for a mean of 24 months (± 8.1SD). Best-corrected visual acuity improved from 0.25 logMar (± 0.2SD) to 0.12 (± 0.10SD; *p* = 0.06) at each patient’s last visit. During laser treatment a darkening of the TCs lumina was achieved in 91.3% of lesions. All these lesions fully resolved, whereas TCs, which showed no darkening of their lumen in OCT persisted and required re-treatment with laser. Additional anti-VEGF injections were indicated in only one eye (8.3%). The darkening of the TCs lumina visible in OCT might provide an image-biomarker that indicates successful coagulation of aneurysmatic lesions. Consequently, a significant functional and morphological improvement with need for anti-VEGF treatment in only one eye, was achieved.

*Information concerning the registration of the trial: date of registration*: 11th of december, 2019. Trial registration number: 107/2019.

## Introduction

Vision loss due to diabetic macular edema (DME) and its treatment have become a major socioeconomic burden^1,2^. For many years fluorescein angiography (FA)-based focal/grid macular laser has been the go-to treatment for patients with DME^3,4^.

However, since the introduction of intravitreal anti-vascular endothelial growth factor (VEGF) therapy, macular laser has lost its role as a primary treatment in patients with center-involving DME. Data from randomized clinical trials examining fluorescein angiography-based central laser therapy (e.g. modified ETDRS laser treatment) in addition to intravitreal anti-VEGF showed no statistically significant benefit compared with intravitreal anti-VEGF monotherapy for visual acuity outcomes^5–8^. The potential risks of laser therapy, such as the development of a choroidal neovascularization, foveal burns or enlargement of laser scars in the absence of an additional functional benefit seem to have underpinned the paradigm of “anti-VEGF injections alone”^9^.

With the introduction of OCT imaging non-invasive visualization of intra- and subretinal fluid became possible, thereby greatly impacting the diagnosis of diabetic macular edema and other retinal disease^10–19^.

However, established standards for focal laser therapy are still based on FA findings and do not include guidance by OCT imaging for the detection of potential laser targets. Furthermore, an increasing amount of recently published data highlights the superiority of indocyanine green angiography (ICGA) compared to fluorescein angiography (FA) for detecting specific microaneurysms that are responsible for the formation of DME^20–23^. It was found that, compared to FA, ICGA depicts microaneurysms more specifically in the area of edema, especially in the late-phase of the angiography^20,21^. In the early phase of the angiogram ICG starts to partially accumulate in specific aneurysms until they become completely filled in the later phases (≥ 10 min). When the dye is washed out of the retinal vessels and other microaneurysms, some lesions show prolonged ICG staining^21,23^.

Because of this distinct staining-pattern it was suggested to term these lesions “teleangiectatic capillaries” (TC) to differentiate them from common microaneurysms^21^. The presence of TCs has also been described in patients with macular edema secondary to retinal vein occlusion and idiopathic macular teleangiectasia^24–26^. There is however very little data on the functional and morphological outcome of ICGA-guided laser therapy of TCs in patients with diabetic macular edema^20^. Our study presents long-term results of an ICGA-guided approach to focal laser therapy. Additionally, we used customized OCT single-scans to evaluate the immediate as well as the consecutive morphological response of the TCs to their direct laser photocoagulation, for which the term “OCT monitoring” will be used in this manuscript.

## Methods

In this retrospective study we evaluated data from 12 eyes of 9 patients with center-involving DME (central subfield thickness (CST) > 300 µm) in whom TCs were selectively targeted with focal laser photocoagulation in the macular region between October 1, 2014 and August 22, 2018. This study was approved by the Institutional Review Board Burgenland (KRAGES—burgenländische Krankenanstalten Gesellschaft m.b.h.). Due to the retrospective nature of the study the Institutional Review Board Burgenland (KRAGES—burgenländische Krankenanstalten Gesellschaft m.b.h.) has waived off the need for informed consent. The study adhered to the tenets of the Declaration of Helsinki. All laser treatments were performed by the same retina expert (PD) in an ophthalmological practice.

### Multimodal imaging and scan planning tool-based treatment planning

**Figure 1:**
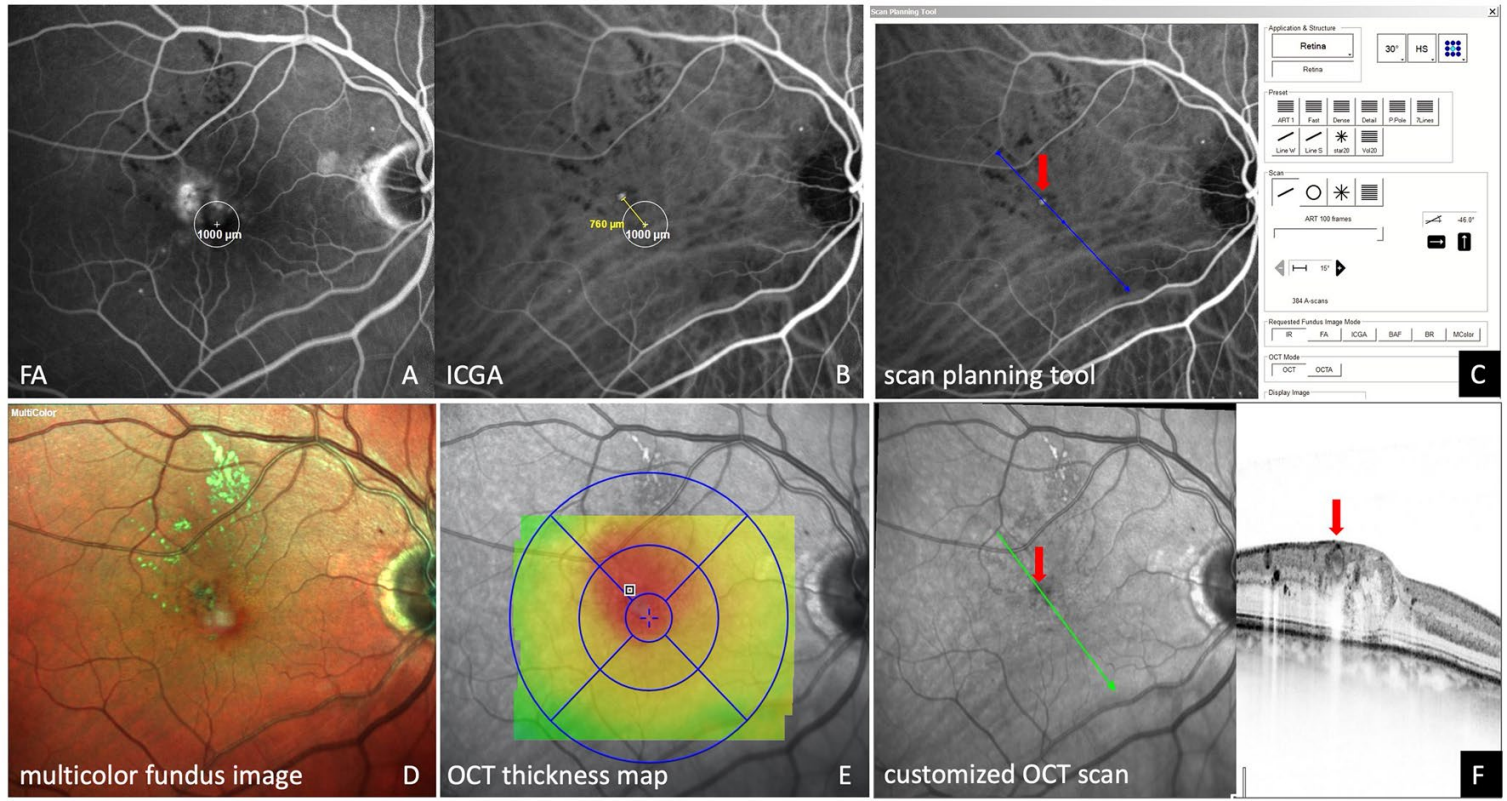
Multimodal imaging of a patient with treatment-naive diabetic macular edema (DME) before indocyanine green angiography (ICGA)-guided and OCT monitored laser therapy. A circle with a radius of 500 µm is positioned in the center of the foveal avascular zone (FAZ) as determined with fluorescein angiography (FA) (**A**). Due to simultaneous acquisition of FA and ICGA images, measurements from FA can accurately be transferred to the ICGA image, thus enabling precise distance measurements from the center of the FAZ to the teleangiectatic capillary (TC) (**B**). Mid- to late-phase ICGA depicts one TC 760 µm from the foveal center. Diffuse leakage is seen in the late-phase of FA in and around this area (**A**). The scan planning tool, provided by the OCT systems software, is used to create a customized OCT B-scan centered on the TC based on the patients’ ICGA images (**C**; blue line/red arrow).The TC is clearly visible in the OCT B-scan, showing a hyporeflective lumen, compared with the surrounding vessel wall (**F**; red arrow). This OCT single scan is used for OCT monitoring of the immediate treatment response during and after laser therapy as well as for long-term follow-up. A multicolor fundus image displays hard exudates superotemporal from the TC (**D**). The retinal thickness map, centered on the fovea, presents asymmetrical distribution of the edema with the peak of the edema near the ICGA positive lesion (**E**). Based on this multimodal image data, the patient was scheduled for focal laser therapy (see Fig. 2).

**Figure 2:**
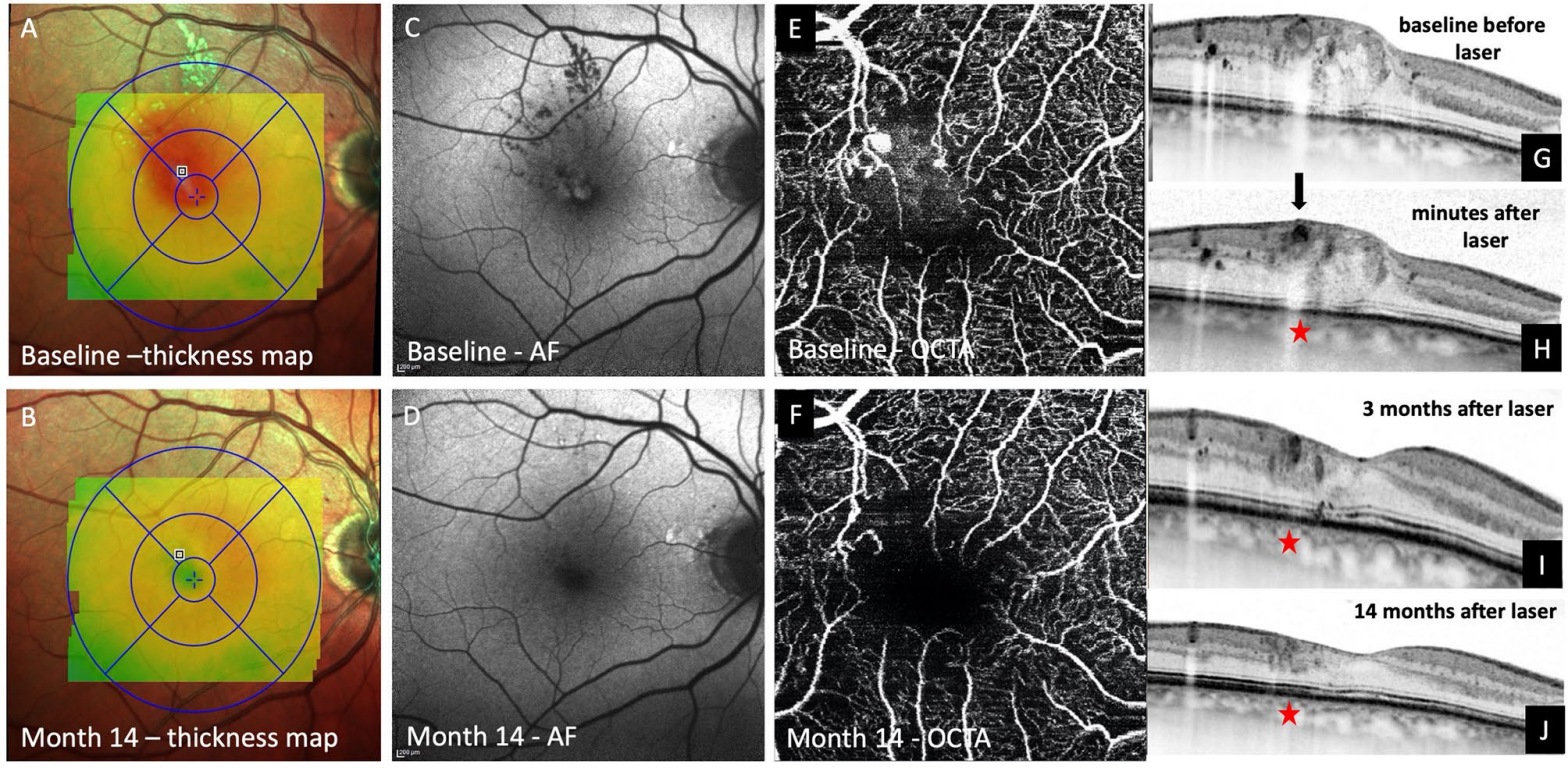
Multimodal image data of the same patient with treatment-naive center involving diabetic macular edema (see Fig. 1). Before laser therapy, pronounced thickening of the retina is seen superotemporal from the fovea (**A**). Fundus autofluorescence (FAF) reveals the presence of hard exudates prior to laser therapy (**C**). OCT angiography (OCTA) shows the same lesion (**E**) as late-phase indocyanine green angiography (ICGA), where one teleangiectatic capillary (TC) was seen prior to laser therapy (Fig. 1B, C). Focal laser therapy is applied to the TC using a laser system operating at a wavelength of 577 nm. A darkening of the TCs lumen is seen in OCT immediately after laser therapy suggestive of immediate closure of the lesion during the laser session (**H**; black arrow). 3 months after laser therapy shrinking of the TC and almost full resolution of the edema were detected with OCT (**I**). Full resolution of the edema (**B**), hard exudates (**D**) and the TC (**J**) with no need for further interventions within a follow-up period of 14 months was achieved. OCTA shows continuing loss of flow signal of the TC 14 months after therapy (**F**). Best-corrected visual acuity increased from 20/30 Snellen visual acuity at baseline before laser therapy to 20/20 Snellen 14 months after laser therapy. Central subfield thickness (CST) decreased from 412 to 299 µm after 6 months and remained stable at 300 µm after 14 months, which was the patient’s last visit within the follow-up period. No changes in the outer retina of this patient are seen in OCT after laser photocoagulation (**D**, **H**). Since only partial coagulation of the upper portion of the TC targeted with the laser was seen, the underlying retinal tissue might have been spared from laser-induced damage.

**Figure 3:**
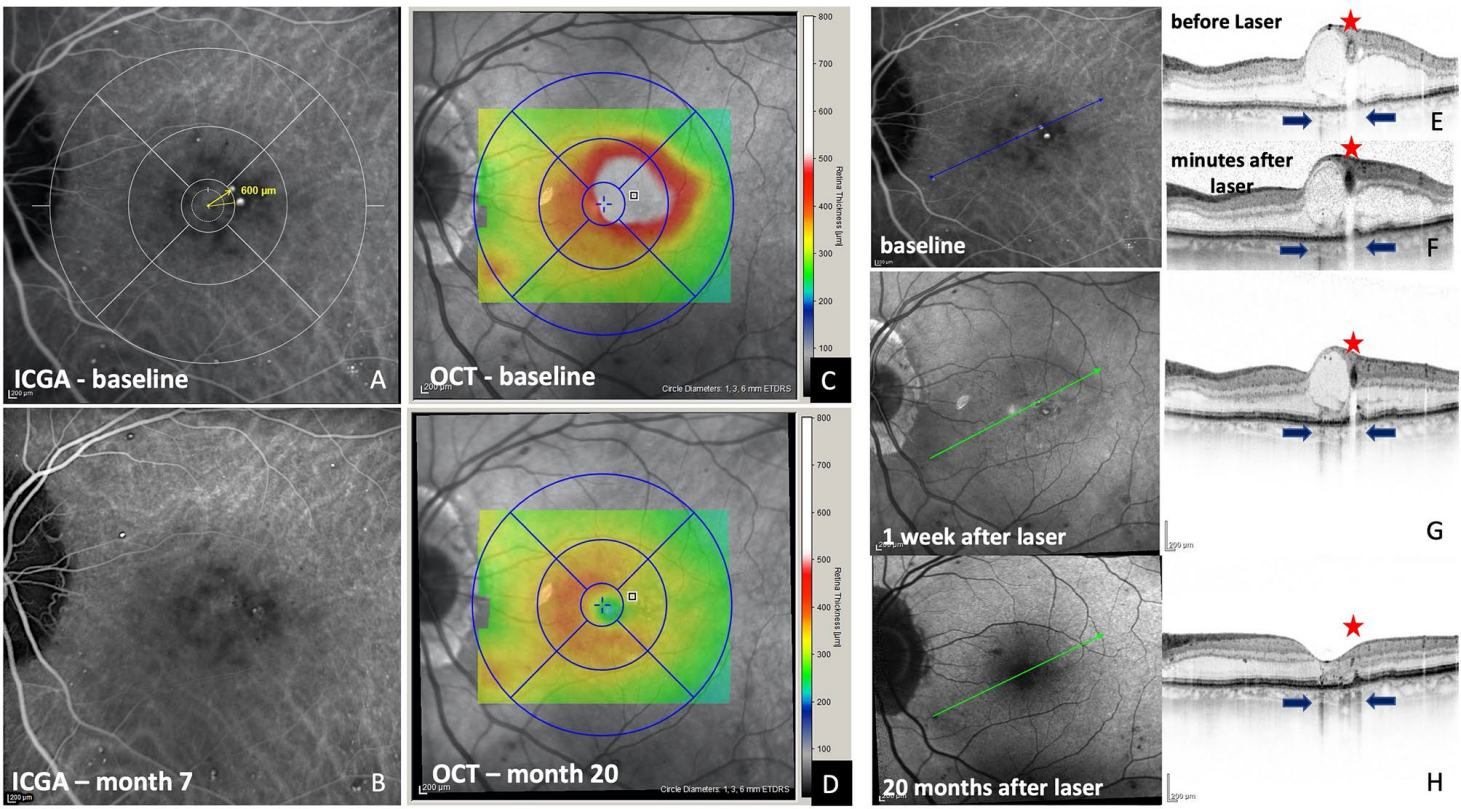
Multimodal imaging of a patient with treatment-naive center-involving diabetic macular edema. At baseline, indocyanine green angiography (ICGA) shows two teleangiectatic capillaries (TCs) at a distance of 600 µm from the foveal center, located right next to the peak of the edema (**A** and **C**). One representative OCT single scan that was created based on the patient’s ICGA images leads right through the center of the more superiorly located lesion (**E**; blue line). A darkening of the TCs lumen is seen in the OCT B-scan as immediate reaction to laser therapy (compare **E** and **F**; red asterisks). The OCT scan acquired at baseline shows a small area of retinal pigment epithelium atrophy due to intermediate age-related macular degeneration before laser therapy right next and also underneath the TC (**E**; blue arrows). No additional retinal damage is seen in OCT one week after successful laser treatment (**F**). The TCs are no longer visible in ICGA, performed 7 months after laser therapy (**B**). Following closure of the TC, OCT reveals a small area of atrophy directly underneath this area. (**G**; right blue arrow). However, due to blockage of the OCT signal by the TC at baseline, it remains unclear if this area of atrophy was present before, or was induced by laser therapy. Best-corrected visual acuity (BCVA) increased from 20/32 Snellen VA at baseline to 20/25 9 months after laser therapy, while central subfield thickness (CST) decreased from 567 µm at baseline to 273 µm at month 9. BCVA and CRT remained stable at 20/25 Snellen and 282 µm, respectively 20 months after laser therapy without additional interventions (**D**).

**Figure 4:**
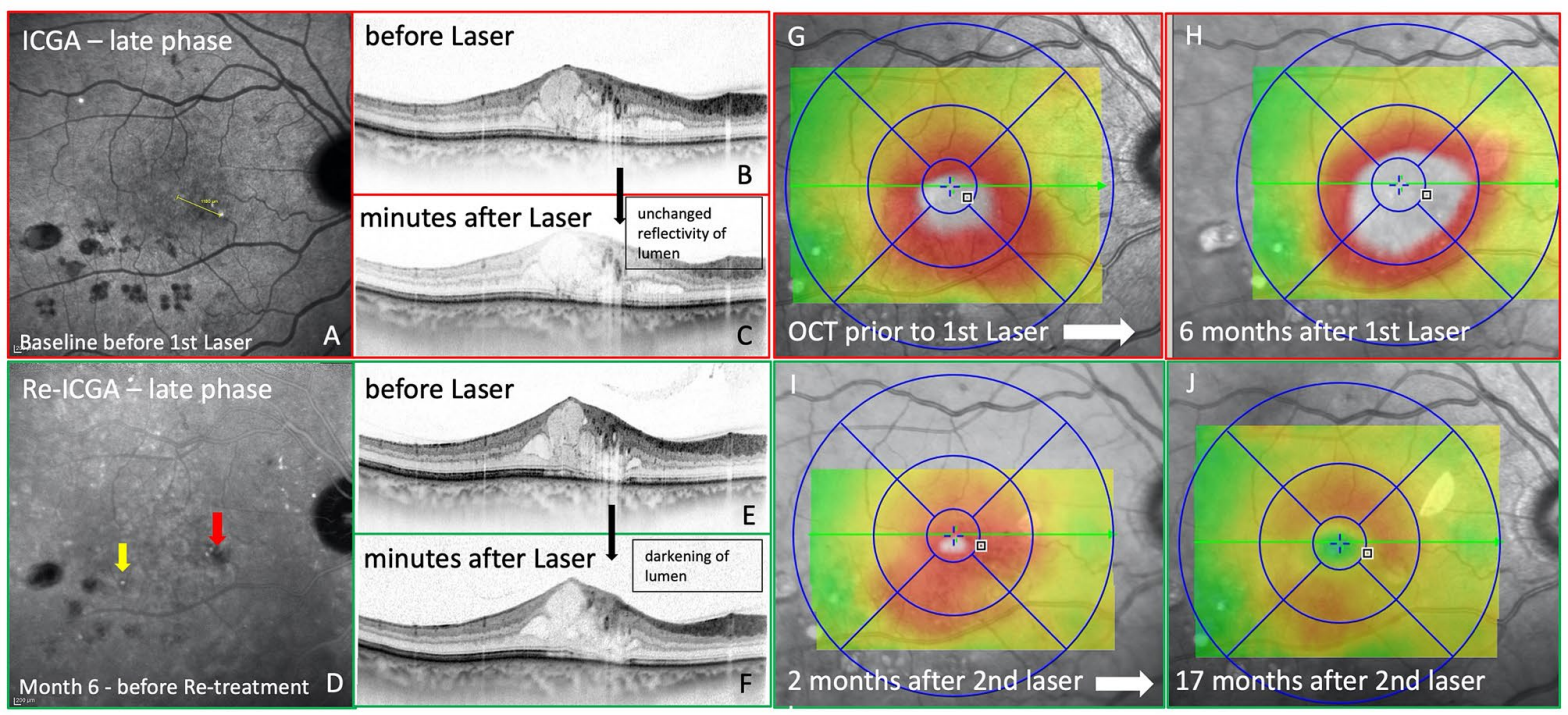
Multimodal imaging of a patient with diabetic macular edema pretreated with anti-VEGF and fluorescein angiography (FA)-based focal/grid laser. Late-phase indocyanine green angiography (ICGA) performed at baseline reveals the presence of two teleangiectatic capillaries (TC) nasally from the fovea (**A**) in near proximity to the peak of the edema (**G**). High resolution OCT single line scans leading right through the center of the larger TC show hyporeflectivity of its lumen, compared to the surrounding vessel wall (**B**). Minutes after the first laser session no change in the TCs lumen was detected (**C**). Consequently, 6 months after laser therapy the TC was still present without a tendency for either shrinking or resolution (**E**). Within the first 6 months after laser therapy the extent of the edema increased (**H**). Therefore, ICGA was repeated, with poor visualization of the TC that was initially targeted with laser, due to partial blocking of the ICG signal by an overlying spot bleeding (**D**; red arrow). However, the OCT scan clearly shows, that the TC is still present (**E**). During the second laser session, a darkening of the TCs lumen was observed with OCT (**F**). Consequently, a marked reduction of the edema was seen as early as 2 months after laser therapy (**I**). 17 months after re-treatment with ICGA-guided focal laser coagulation only little parafoveal retinal swelling remained without central involvement of the edema. (**J**) No additional treatment with anti-VEGF was needed in this patient. Visual acuity increased from 20/32 Snellen visual acuity at baseline to 20/20 23 months after the intial laser therapy with no need for any anti-VEGF injections.

**Figure 5:**
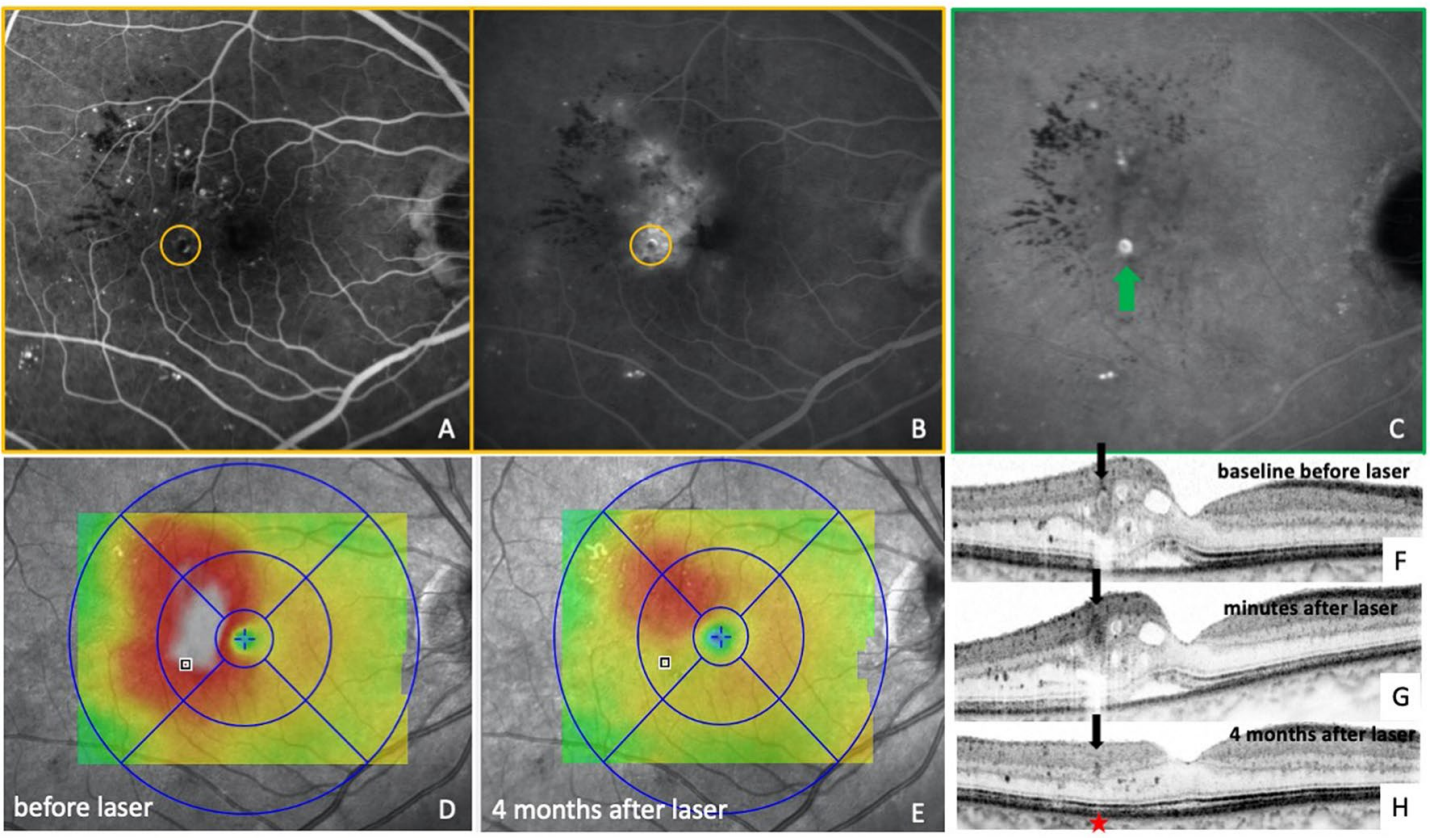
Multimodal imaging of the right eye of a patient with treatment naive diabetic macular edema. Early-phase fluorescein angiography (FA) shows numerous microaneurysms predominantely in the temporal macula (**A**). Late-phase FA reveals an area of diffuse leakage in this area. In contrast, late-phase indocyanine green angiography (ICGA) (≥ 10 min) reveals one bright hyperreflective spot in and around this area. The OCT thickness map revealed extensive diabetic macular edema involving the fovea (**D**). Therefore the patient was scheduled for ICGA-guided focal laser therapy. Note that the OCT single scan leads right through the center of the TC, which shows a hyporeflective lumen compared to the surrounding vessel wall (**F**). Minutes after several laser burst have been applied on the surface on the TC, a darkening of its lumen can be observed with OCT (**G**). Consequently, the TC fully resolved as early as 4 months after the treatment without damage to the underlying photoreceptors (**H**, red asterisk), together with most of the intraretinal fluid and only little remaining parafoveal retinal swelling (**E**).

In patients with center-involving DME, FA and ICGA were simultaneously performed to enable measurements to be transferred from ICGA to FA images and vice versa (Figs. 1A, B, 4A). Targeted laser therapy was applied at TCs at a distance of > 500 µm from the foveal center. Prior to laser treatment, customized OCT scans were created based on the eyes’ ICGA images using a scan planning tool provided by the OCT systems software (Spectralis HRA + OCT2; Spectralis; Heidelberg, Germany). Single OCT line scans were positioned directly on the center of the TCs (Fig. 1C, F, 3E–H, 5F). The automated real-time tracking (ART) function of the OCT device, which enables averaging of up to 100 scans from the exact same location, was used to acquire scans of optimum quality before, during, immediately after laser treatment and at each follow-up visit (Fig. 2G–J, 3E–H). The follow-up function of the OCT device was used to facilitate repeated scan acquisition during the laser treatment and at the patients’ regular visits during the follow-up period. Additionally, a macular OCT scan was performed at each visit using the following settings: 20° × 15° macular grid centered on the fovea, 29 scans, ART 10–15, high resolution (Fig. 1E; 2A, B; 3C, D; 4G–J, 5D–E).

### ICGA-guided laser treatment

FA and ICGA images of the patients were displayed on a monitor directly next to the laser device to facilitate localization of the TCs during the procedure. Laser treatment was performed using a laser system operating at a wavelength of 577 nm (Pascal Streamline, Topcon Medical Systems Inc., Oakland, New Jersey) with the following settings: impulse duration 20 ms, spot size 60–100 µm, 100–350 mW, central mirror of Goldmann contact lens. Methocel 2% contact gel was administered prior to insertion of the contact lens. Laser bursts were applied directly on the surface of the TCs until a minimal greyish-white discoloration of these lesions was seen. After this initial treatment-phase the customized OCT scans, placed directly through the TCs, were used to monitor changes in the reflectivity of their lumina. As soon as a darkening of a TCs lumen was seen in the OCT single scans no further laser bursts were applied to this lesion (compare 2G and H; 3E and 3F; 4E and 4F; 5F and G). The repeated acquisition of the customized OCT single scans during laser treatment was usually performed one to three times until a darkening of the TCs lumina was visible. In the ophthalmological practice where all laser treatments were performed, the laser machine is positioned in a 90° angle in relation to the OCT device. Thus, the patient can remain seated by turning 90° to the side, which facilitated time efficient acquisition of the repeated OCT single scans during laser treatment.

### Study measures

BCVA was assessed using 6 m Snellen visual acuity charts. OCT was routinely performed at each visit during the follow-up period. CST was measured in the central field of the macular ETDRS grid (Fig. 1E, 2A, B, 3C, D, 4G–J). Multicolor fundus and/or fundus autofluorescence images were acquired to document the presence and resolution of hard exudates throughout the follow-up period (Fig. 1D, 2A–D). Further, OCTA was used in selected patients to non-invasively monitor successful loss of the TCs flow signal throughout the follow-up period (2E–F).

### Statistical analysis

The comparison of BCVA and CST values between baseline and month 6–8 as well as month 12–15 and the last visit before November 30, 2019 were performed by mixed models (SAS Proc mixed). For statistical analysis BCVA data was converted from Snellen VA to the logarithm of the minimum angle of resolution (logMAR), The number of degrees of freedom was calculated using the Kenward–Roger-approximation. The difference to baseline was the dependent variable. Patient was taken as a random factor. For analysis of month 6–8 and month 12–15, respectively, only the intercept was included in the model, for analysis of the last visit, the follow-up time (centered at a mean follow-up time of 23.75 months) was additionally included. An estimate for the mean difference to baseline with 95% confidence limits and the *p*-value (H0: mean difference to baseline is equal to zero) were calculated. Statistical analyses were calculated with SAS 9.4 and R 4.0.5. The significance level was set to alpha = 0.05.

### Descriptive statistics

We calculated the average duration until shrinking and resorption of the TCs was seen during follow-up. Further, two graders (F.D and A.D) independently evaluated the reflectivity of the TCs lumina in the OCT single scans that were acquired before and during laser therapy. Reflectivity of the lumen was graded as hypo- or hyperreflective, compared to the surrounding vessel wall (Figs. 2G, H, 3E, F, 4B, C, E, F, 5F, G) or as ungradable. For this evaluation the scans were presented to the graders in random order without information whether a scan was acquired before or after laser therapy. In a second step they evaluated the change in the lumen’s reflectivity of the last single scan that was acquired during laser therapy compared to the scan acquired prior to laser therapy. The TCs lumina’s reflectivity was graded as darkened, unchanged or not gradable.

Additionally, we measured the distance from the maximum height of the edema at baseline to the largest TC in µm and we determined the mean number of laser targets treated per eye, the mean distance from the fovea to the laser target closest to the fovea, the mean size of the TCs treated with laser and the number of retreatments with laser as well as the proportion of patients requiring intravitreal injections of anti-VEGF in addition to laser therapy.

## Results

### Patients

The mean age of the patients (3 female, 6 male) was 71 years (± 9.6 SD), ranging from 61 to 84. The mean follow-up was 24.0 months (± 8.1 SD), ranging from 11 to 43 months. Prior to ICGA-guided laser photocoagulation 6 of the 12 eyes were pretreated with anti-VEGF. 4 of the 6 pretreated eyes additionally had FA-based focal/grid laser photocoagulation prior to anti-VEGF therapy and the other 2 pretreated eyes received anti-VEGF only. 6 eyes were treatment naive. In 5 patients (8 eyes) HbA1c values were available before laser therapy as well as during the follow up. Mean HbA1c before laser therapy was 6.8% ranging from 6.3 to 7.0 and 7.01% after laser therapy ranging from 6.4 to 7.2.

### Functional and morphological outcome

**Figure 6:**
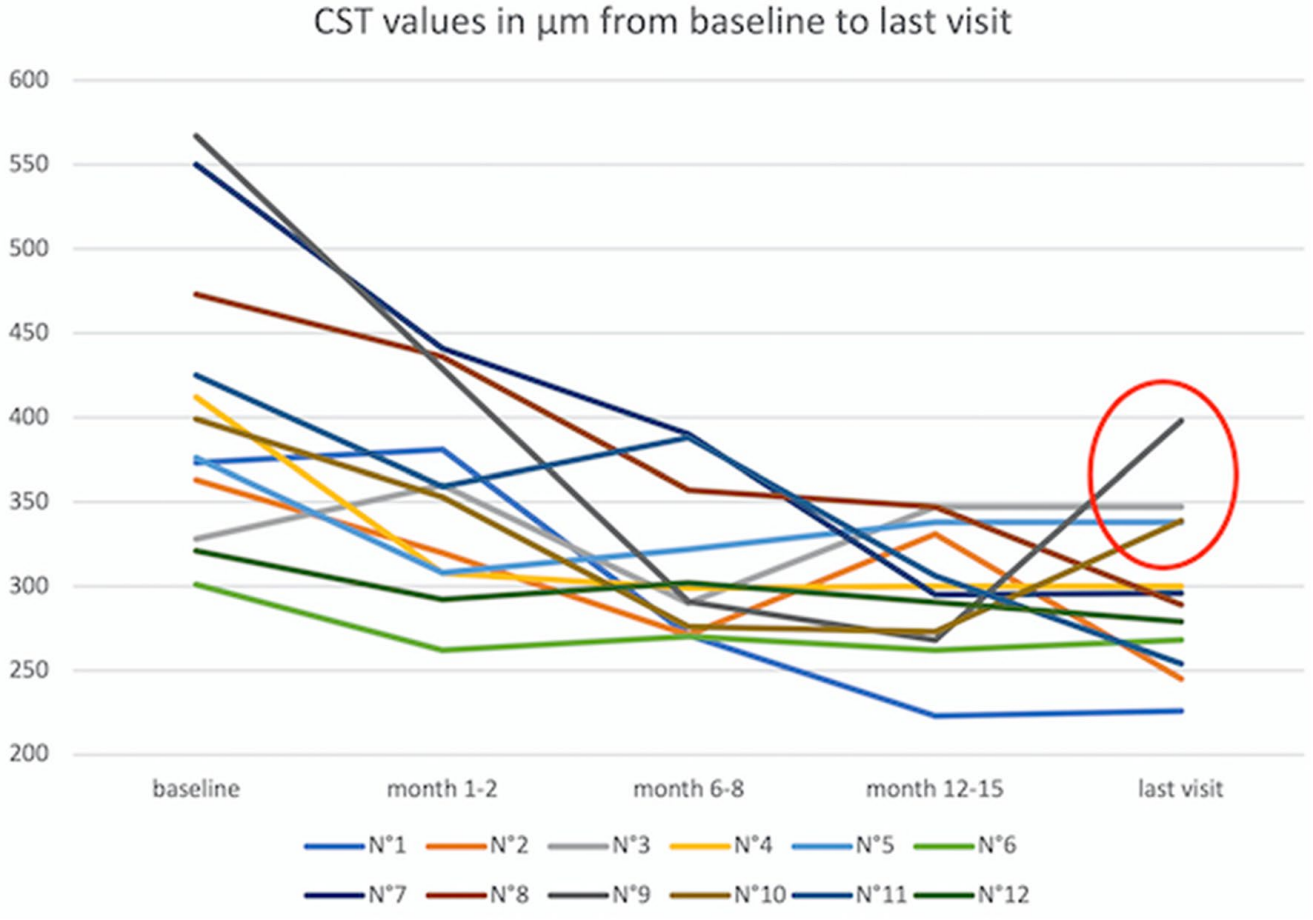
Morphological results of all eyes including the first visit after laser within the first 2 months. 8 eyes show CST-values below 300 µm throughout the follow-up period. Note that 4 eyes have CST values above 300 µm at the end of the follow-up (encircled in red). 2 of these 4 eyes (N°9 and N°10) had re-treatment with laser at their last visit during the follow-up period. One of the other 2 eyes showed progression of an epiretinal membrane, that was already present at baseline and presented with diffuse retinal swelling and no intraretinal cysts (N°3). The other eye showed stable visual acuity and an increase in CST of 30 µm from month 6 to 28 months after a good initial morphological response to laser therapy and was therefore not re-treated neither with focal laser nor with intravitreal anti-VEGF.

The mean BCVA at baseline was 0.25 logMar (± 0.2 SD; 95%CI 0.14–0.35) and improved to 0.19 logMar (± 0.17 SD; 95%CI 0.10–0.28) at months 6–8 and to 0.13 logMar (± 0.10 SD; 95%CI 0.08–0.19) at months 12–15. BCVA at the patients last visit was 0.12 (± 0.10 SD; 95%CI 0.06–0.17). BCVA improvements were statistically significant from baseline to month 6–8 (*p* = 0.05, mixed model), from baseline to month 12–15 (*p* = 0.02) as well as a tendency towards statistical significance from baseline to each last visit of the patients (*p* = 0.06). CST at baseline was 418 µm (± 85 µm SD; 95%CI 370–466) and decreased to 349 µm (± 143SD; 95%CI 260–438) after a mean of 1.3 months after laser therapy. Mean CST further decreased statistically significantly to 311 µm (± 44 µm SD; 95%CI 285–335) at months 6–8 (*p* = 0.003, mixed model) and remained stable at 299 µm (± 23 µm SD; 95%CI 270–326) at months 12–15 (*p* = 0.001) and at 298 µm (± 50 µm SD; 95%CI 265–317) at the last visit (*p* = 0.001). Morphological and functional results for each patient are presented in Table 1 and Fig. 6. The mean maximum height of the edema at baseline was 484 µm (± 74 µm SD). No symptomatic atrophy, foveal burns or development of a choroidal neovascularization secondary to laser treatment were seen in any of the eyes during the follow-up period.

**Table 1:** Overview of best-corrected visual acuity (BCVA) results in Snellen decimals, the individual follow-up time in months and the re-treatments required during the follow-up period of all eyes.

	BSL	Mo6-8	Mo12-15	Last visit		Mean follow-up in months	Additional treatment
N°1	0.8	0.8	0.8	1.0		23	Re-laser
N°2	0.16	0.2	0.25	0.6		24	Re-laser
N°3	0.4	0.6	0.6	0.6		43	–
N°4	0.6	0.8	1.0	1.0		14	–
N°5	0.6	0.8	0.8	0.6		28	–
N°6	0.8	0.6	0.6	0.6		30	–
N°7	0.6	0.8	1.0	1.0		17	–
N°8	0.8	0.8	0.8	1.0		24	–
N°9	0.6	0.6	0.8	0.8		29	Re-laser
N°10	0.4	0.6	0.6	0.6		18	Re-laser
N°11	0.8	0.8	0.8	0.8		24	4 × anti-VEGF
N°12	0.8	0.8	X	0.8		11	Re-laser

### Characteristics of teleangiectatic capillaries (TCs)

The mean number of TCs targeted with laser was 1.5 per eye (± 0.8 SD; 95%CI 1.0–2.0), ranging from 1 to 3. The mean distance from the largest TC to the maximum height of the edema was 413 µm (± 218 SD) ranging from 51 to 743 µm. The mean size of the TCs was 170 µm (± 39 µm SD; 95%CI 156–184). The mean distance of the TCs closest to the fovea was 960 µm (± 441 µm SD; 95%CI 711–1209), ranging from 549 to 2039 µm. According to the analysis of both graders prior to laser therapy all TC’s presented with a hyporeflective lumen compared to the surrounding vessel wall in the OCT single scans (1F, 2G, 3E, 4B, E, 5F). During the first laser treatment an immediate darkening of the TCs lumina could be monitored with OCT in 10 out of 12 eyes (Figs. 2H, 3F, 4F, 5G). In total 17 laser sessions, including the 12 initial treatments as well as the 5 re-treatments, were performed. The analysis performed by 2 independent graders showed hyporeflectivity of the lesions lumina in the same 19 out of 22 TCs (91.3%) as immediate response to laser treatment, whereas 3 TCs in 2 eyes showed no changes in reflectivity of their lumina and 2 TCs were too small and could therefore not be evaluated.

Shrinking of all TCs, in which the immediate darkening of their lumina was observed, was seen in OCT after a mean of 1.3 months ranging from 1 week to 2 months (Figs. 2I, 3G). Full resolution of all these lesions was seen after a mean follow-up of 4 months ranging from 1 to 7 months (Figs. 2J, 3H; red asterisk).

### Additional treatment

In the 10 eyes where a darkening of the TCs lumina was seen immediately after laser treatment, no additional laser therapy was necessary within the first year of follow-up (Figs. 2, 3). In the other two eyes, both presenting with one TC, no darkening of the TCs lumina was observed in OCT during the first laser treatment (4B and C). Additional ICGA-guided laser therapy was therefore necessary in both eyes due to persistence of the TCs (compare Fig. 4B, D, E) without a tendency for either shrinking or resorption of these lesions and increasing accumulation of intraretinal fluid 6 months after the initial treatment (compare Fig. 4G, H). During re-treatment of these 2 eyes with laser, an immediate darkening of the TCs lumina was then seen in the OCT single scans (compare Fig. 4E, F). Consequently, both TCs completely resolved after 2 and 5 months respectively together with the associated intraretinal fluid. In two eyes of one patient additional laser therapy was necessary due to re-growth of a TC at the same location 18 months (right eye) and 26 months (left eye) after the first laser treatment. In one eye re-treatment with ICGA-guided laser was necessary due to growth of a new TC at a different location with accompanying intraretinal fluid accumulation 20 months after the initial laser therapy. Within the follow-up period four intravitreal injections of aflibercept were indicated in only 1 out of 12 eyes (8.3%). No additional treatment with intravitreal anti-VEGF was necessary in the remaining 11 eyes.

## Discussion

Our results show the potential of ICGA-guided focal laser therapy in selected patients with pretreated as well as treatment naive center-involving DME. In contrast to established FA-based laser protocols, in particular the modified ETDRS laser treatment, our approach is minimally invasive in its nature, with a mean of only 1.5 TCs treated per eye^27^. Despite the very low number of laser targets, there was a statistically significant improvement in BCVA at month 6–8 and month 12–15 and a strong tendency towards statistical significance at each patients last visit.

At month 6–8 a statistically significant decrease in mean CST by more than 100 µm was achieved, that could be maintained at month 12–15 as well as at the last visit, with minimal need for additional intravitreal injections of anti-VEGF in only 1 out of 12 eyes. Thus, our results suggest that ICGA-guided photocoagulation of TCs could vastly reduce the need for intravitreal anti-VEGF injections in patients with center-involving DME. In contrast to previously published studies on ICGA-guided laser therapy, treatment naive patients were also included in this study^7,10^. Thus, our results indicate that there might be a subtype of DME, that profits from initial ICGA-guided laser therapy with no need for additional intravitreal anti-VEGF injections in the vast majority of eyes during a mean follow-up of almost 2 years. Interestingly, a marked reduction in CST was already seen within the first two months after a mean follow-up of about 7 weeks. This early morphological improvement and the shrinking of the TCs, observed after a mean of 1.3 months was followed by the full resolution of all these lesions. Additionally, in eyes in which an immediate darkening of the TCs lumina was seen, the edema resolved almost completely, whereas in the two eyes where no darkening of the lesions lumina was observed after the first laser session, the TCs and the edema persisted and the patients therefore required retreatment with laser.

Hence, our results show that OCT monitoring of the treatment response provides an image-based endpoint, that is indicative of the immediate successful closure of an aneurysmatic lesion. This direct visual feedback can be evaluated by the ophthalmologist while the patient is still in the clinic and laser therapy can be continued immediately step by step until a darkening of the lesion's lumen is seen in OCT. Therefore, OCT monitoring might increase the rate of aneurysm closure compared to biomicroscopic or fundusphotographic evaluation alone^22^.

Further, the evaluation of the reflectivity of the TCs lumina by two independent graders demonstrated good reproducibility of this imaging biomarker.

It is known from randomized clinical trials, that fluorescein angiography-based focal laser therapy in addition to intravitreal anti-VEGF did not result in an additional functional benefit compared to anti-VEGF monotherapy^5–8^.

With the introduction of OCT imaging non-invasive visualization of intra- and subretinal fluid became possible, thereby greatly impacting the diagnosis of diabetic macular edema and other retinal disease^10–19^.

However, current standards for focal laser therapy do not include OCT imaging, neither for the detection of lesions nor for monitoring aneurysm closure. Also, they do not include indocyanine green angiography for the detection of potential laser targets^28–30^.

A big advantage of OCT compared to dye-based angiography is, that it is able to depict the amount of fluid in and below the retina. Thus, with the combination of dye-based angiography and OCT we were able to obtain information about the contribution of specific lesions to retinal fluid accumulation.

In accordance with previously published studies we found, that ICGA seems to be particularly useful to detect highly exudative aneurysms, since in our study the largest TC of each patient was located in close proximity to the maximum height of the edema, which further highlights the close connection between these lesions and their role in the formation of DME^20,23^. Hence, our results indicate that not randomization, but specific selection of eyes based on the presence of TCs might be necessary to detect those eyes that indeed profit from focal laser therapy.

Further, OCT monitoring of the immediate treatment response may reduce the number of laser retreatments needed to achieve successful coagulation of lesions, which would increase the efficacy of focal laser therapies. Especially for larger lesions, which are known to be more difficult to occlude, OCT monitoring can certainly prove beneficial^31^. What is more, in some of our patients, successful closure of TCs could be achieved without damage to the underlying photoreceptors or changes in the retinal pigment epithelium visible in OCT, while achieving full resolution of the edema (Figs. 2J, 5H). This highlights the potential of OCT monitoring to minimize laser induced retinal damage, thus possibly reducing potential side effects of focal laser therapy such as enlargement of laser scars or the development of a choroidal neovascularization.

OCT angiography (OCTA), a recent advancement in ophthalmic imaging, allows non-invasive detection of blood flow in and around the macular region. Its utility to detect TCs has been evaluated in a series of 20 patients undergoing both ICGA and OCTA examination. It was found that TCs showed rather poor visibility in OCTA, possibly due to presence of intraluminal material inside the TCs, that reduces the flow velocity below the threshold of the OCTA device so that they are no longer detectable^21^.

Ultra-widefield imaging, another innovation in retinal imaging, allows for a standardized evaluation of diabetic retinal changes up until the far periphery^32,33^. While resolution of ultra-widefield imaging devices is useful to detect peripheral ischemia and neovascularization and still might be sufficient to depict TCs, we know from clinical experience, that small retinal vessels in the macular region are commonly not well visualized. This makes the exact localization of the TCs more difficult, since those small retinal vessels serve as reference points to locate the TCs during laser therapy. This is particularly important in patients with a lot of microaneuryms but only few TCs. What is more, TCs that are involved in the formation of center involving DME are rarely located outside the 30° field of view imaging which can be performed with the Spectralis Heidelberg HRA + OCT device^20,21^. Additionally the scan planning tool available for this device allowed us to create the OCT single scans that we used to monitor the darkening of the TCs lumina.

In times of emerging automation and navigated laser systems, implementing OCT monitoring in such devices to enable the immediate evaluation of the treatment response automatically would be of great interest to further optimize the outcome, safety and accuracy of focal laser therapies in patients with DME.

The automated real-time tracking function of the OCT device, which enables up to 100 OCT scans, acquired at the exact same location, to be averaged, was crucial for obtaining images of sufficient quality to assess changes in the reflectivity of the TCs lumina. Thus, intraprocedural monitoring of the treatment response was possible, despite the use of a contact agent, as required for focal laser therapy with a contact lens. Combining OCT monitoring of the treatment response with an OCT scan planning tool software allowed the post hoc customization of OCT scans, that are based on the patient’s ICGA images, any time after completion of the ICGA examination. Thus, the operator of the device does not necessarily have to acquire OCT scans localized exactly at the spots of interest during the ICG examination, which is often challenging in routine clinical practice. Post hoc scan planning further reduces the number of scans needed to depict all potential laser targets, because at least two lesions can be imaged with a single OCT scan. This allows for even more time efficient intraprocedural monitoring of the treatment response, making it feasible for clinical practice.

Our laser protocol is most similar to that of a pilot study published by Paques et al., in which four eyes of three patients with DME were followed up for 6 months after ICGA-guided laser therapy of TCs^20^. They applied focal laser treatment to TCs larger than 150 µm in eyes with persistent DME after treatment with anti-VEGF. However, their patients differed from ours in terms of baseline BCVA. The mean BCVA at baseline was 20/32 Snellen VA in our patients and 20/200 in theirs. In their study, BCVA improved in 2 eyes and remained stable in another 2 eyes, while all eyes showed morphological improvement without additional therapy during a follow-up of 6 months. In our patients BCVA improved in 8 eyes, remained stable in 3 eyes and worsened only in 1 eye. Morphological improvement was achieved in all of our patients. In their patients mean CST at baseline was 515 µm and the mean number of TCs was 3.4^20^. In our patients mean CST at baseline was 418 µm and the mean number of TCs was 1.5. These differences suggest a connection between more severe DME and a higher number of TCs per eye.

Another approach highlighted the functional and morphological benefit of ICGA-guided photocoagulation of microaneurysms, but not TCs in particular, using an automated navigated laser photocoagulation system (Navilas,OD-OS Gmbh, Teltow, Germany) in 8 eyes of 6 patients with DME pretreated with anti-VEGF^22^. However, despite a mean of 22 laser targets per eye, compared with 1.5 targets in our patients, the reduction in CST they achieved was similar to the results of our study. This suggests, that there are certain ICG-positive aneurysms, probably TCs, that are more relevant to edema formation than others^20,22^. What’s more, 63% of their patients required retreatment with laser after 3 months, compared with 17% in our patients within the first year^22^. This indicates that funduscopic evaluation of the lesion closure, as available in current automatically navigated laser devices, could be misleading. Further, careful selection of specific, possibly larger lesions, that show staining in the late phase (> 10 min) of the ICGA, instead of targeting all lesions visible in earlier phases of the angiography, could lead to a similar functional and morphological outcome. Aneurysm size may be one factor responsible for the amount of exudation, as laser therapy showed only a modest effect in clinical trials that mainly included aneurysms of 150 µm or smaller^34^. Apart from the lesion size, late ICGA staining seems to distinguish highly exudative lesions from others^20,21,23,24^. Nevertheless, other contributing factors, such as the wall thickness or the presence of intraluminal material to the amount of exudation caused by the individual lesion should be investigated in more detail to distinguish highly exudative ICG-positive lesions from others, and to optimize the treatment effect while minimizing the damage inflicted to the retina.

The reason why ICGA is able to depict lesions that are responsible for edema formation, might lie in its chemical properties. It is hypothesized based on histology studies that ICG, because of its amphiphilic properties, binds to hydrophobic intraluminal material such as fibrin, lipids or red blood cell components, which are suggestive of chronic leakage out of these lesions^35^. Fluorescein, which is hydrophilic, would be blocked by such intraluminal material. Interestingly, some of these ICG-positive lesions are not detectable or become only partially filled in FA due to blockage of fluorescein by some intraluminal material. These incompletely filled aneurysms are best visualized in the early phase of FA^25,36^. Adaptive optics scanning light ophthalmoscope (AOSLO) fluorescein angiography combined with AOSLO reflectance images confirmed that partial filling of aneurysms is seen due to the presence of intraluminal material^37^. What is more, some TCs are not visible in early- and late-phase FA and might therefore easily be overlooked without additional ICGA (Fig. 5A–C, orange circles and green arrow).

The limitations of our study are its retrospective nature and the relatively low number of patients. However, to the best of our knowledge, this is the largest study with a long follow-up time on this topic to date.

Depiction of TCs with ICGA, combined with the possibility to systematically monitor the immediate treatment response of laser targets during laser treatment with OCT, as shown in our study, encourages a re-evaluation of the role of focal laser therapy in patients with center-involving DME. Additionally, there is some evidence that the presence of late-phase ICG positive aneurysms, but not FA positive aneurysms, is associated with recurrent DME under anti-VEGF therapy^38^.

However, there is still need for further studies investigating the connection between the anti-VEGF treatment response and the presence of TCs. A large clinical trial comparing the outcome of intravitreal injections of anti-VEGF with and without additional ICGA-targeted laser, including OCT monitoring of the immediate treatment response, would be of utmost importance to better understand the role of ICG-guided central macular laser for the treatment of patients with center-involving DME.
